# Characteristics of delirium and its association with sedation and in-hospital mortality in patients with COVID-19 on veno-venous extracorporeal membrane oxygenation

**DOI:** 10.3389/fmed.2023.1172063

**Published:** 2023-05-25

**Authors:** Philip Young-woo Sun, Jonathon Fanning, Anna Peeler, Benjamin Shou, John Lindsley, Giorgio Caturegli, Glenn Whitman, Stephanie Cha, Bo Soo Kim, Sung-Min Cho, HERALD investigators

**Affiliations:** ^1^Division of Neurosciences Critical Care, Departments of Neurology, Neurosurgery, and Anesthesiology and Critical Care Medicine, Johns Hopkins University School of Medicine, Baltimore, MD, United States; ^2^Division of Cardiothoracic Surgery, Department of Surgery, Johns Hopkins Hospital, Baltimore, MD, United States; ^3^King's College London, Cicely Saunders Institute of Palliative Care, Policy, and Rehabilitation, London, United Kingdom; ^4^Johns Hopkins University School of Medicine, Baltimore, MD, United States; ^5^Department of Pharmacy, Johns Hopkins Hospital, Baltimore, MD, United States; ^6^Department of Surgery, Yale School of Medicine, New Haven, CT, United States; ^7^Department of Anesthesiology and Critical Care Medicine, Johns Hopkins University, Baltimore, MD, United States; ^8^Department of Pulmonary Critical Care Medicine, Johns Hopkins University, Baltimore, MD, United States

**Keywords:** extracorporeal membrane oxygenation, delirium, deep sedation, analgesia, hospital mortality

## Abstract

**Background:**

Veno-venous extracorporeal membrane oxygenation (VV-ECMO) has been used in patients with COVID-19 acute respiratory distress syndrome (ARDS). We aim to assess the characteristics of delirium and describe its association with sedation and in-hospital mortality.

**Methods:**

We retrospectively reviewed adult patients on VV-ECMO for severe COVID-19 ARDS in the Johns Hopkins Hospital ECMO registry in 2020–2021. Delirium was assessed by the Confusion Assessment Method for the ICU (CAM-ICU) when patients scored−3 or above on the Richmond Agitation-Sedation Scale (RASS). Primary outcomes were delirium prevalence and duration in the proportion of days on VV-ECMO.

**Results:**

Of 47 patients (median age = 51), 6 were in a persistent coma and 40 of the remaining 41 patients (98%) had ICU delirium. Delirium in the survivors (*n* = 21) and non-survivors (*n* = 26) was first detected at a similar time point (VV-ECMO day 9.5(5,14) vs. 8.5(5,21), *p* = 0.56) with similar total delirium days on VV-ECMO (9.5[3.3, 16.8] vs. 9.0[4.3, 28.3] days, *p* = 0.43). Non-survivors had numerically lower RASS scores on VV-ECMO days (−3.72[−4.42, −2.96] vs. −3.10[−3.91, −2.21], *p* = 0.06) and significantly prolonged delirium-unassessable days on VV-ECMO with a RASS of −4/−5 (23.0[16.3, 38.3] vs. 17.0(6,23), *p* = 0.03), and total VV-ECMO days (44.5[20.5, 74.3] vs. 27.0[21, 38], *p* = 0.04). The proportion of delirium-present days correlated with RASS (r = 0.64, *p* < 0.001), the proportions of days on VV-ECMO with a neuromuscular blocker (r = −0.59, *p* = 0.001), and with delirium-unassessable exams (r = −0.69, *p* < 0.001) but not with overall ECMO duration (r = 0.01, *p* = 0.96). The average daily dosage of delirium-related medications on ECMO days did not differ significantly. On an exploratory multivariable logistic regression, the proportion of delirium days was not associated with mortality.

**Conclusion:**

Longer duration of delirium was associated with lighter sedation and shorter paralysis, but it did not discern in-hospital mortality. Future studies should evaluate analgosedation and paralytic strategies to optimize delirium, sedation level, and outcomes.

## Introduction

Delirium, which is characterized by acute and fluctuating alterations in consciousness, often precipitated by underlying medical illness (1), is the most common neurological complication observed in intensive care units (ICUs), with a reported prevalence of up to 82% in mechanically ventilated patients (2). Delirium prevalence has been studied in critically ill patients, including those with post-operative complications (3), general ICU needs (2, 4, 5), acute respiratory distress syndrome (6) (ARDS), extracorporeal membrane oxygenation (ECMO) (7) support, intracerebral hemorrhage (8), status epilepticus (9), and acute heart failure (10). In many of these studies (2, 4, 8, 10), delirium portended worsened patient survival. While there is growing evidence purporting the association between heavy sedation and poor outcomes, findings regarding delirium's role have been inconsistent, and it remains incompletely understood in patients on ECMO support (11–13).

Neurological manifestations of patients with COVID-19 in the ICU have been studied with various methods. In a prospective, multicenter study using the International Severe Acute Respiratory and emerging Infection Consortium (ISARIC) cohort, altered consciousness was found in 10.8% of critically ill patients and was associated with seizures and stroke, and both of these conditions had increased odds of occurrence in patients receiving ECMO (14). Two recent multicenter, retrospective studies on patients with COVID-19 revealed ICU delirium prevalence rates of 61.9 and 54.9% (15, 16). Currently, no study has reviewed the prevalence and characteristics of delirium, as well as its association with analgosedation practice in patients with COVID-19 supported with veno-venous ECMO (VV-ECMO).

Our objectives are to (a) examine the prevalence and duration of delirium in severe COVID-19 ARDS patients on VV-ECMO, and (b) compare the use of ICU medications and hospital course between survivors and non-survivors to identify any potential roles of delirium and heavy sedation on in-hospital mortality. We hypothesized that non-survivors would suffer from delirium more frequently and for longer durations and have a deeper level of sedation, including iatrogenic (i.e., oversedation) and neurological/non-neurological medical complications (i.e., refractory COVID-19 and acute brain injury).

## Methods

### Study design

We retrospectively reviewed 47 consecutively enrolled adult patients on VV-ECMO for severe COVID-19 ARDS, as per the Berlin Definition (17), admitted to the Cardiovascular Surgical Intensive Care Unit (CVSICU) of Johns Hopkins Hospital in 2020–2021. The practicing intensivists followed the ECMO to Rescue Lung Injury in Severe ARDS (EOLIA) Trial entry criteria when making ECMO cannulation decisions (18). All the cannulated patients were included in the Johns Hopkins Hospital ECMO (JHH-ECMO) registry and relevant admission and clinical information was recorded. Patients were included in the study if (a) they were adults (defined as 18 years of age or above) and (b) VV-ECMO cannulation lasted for more than 24 h. Exclusion criteria are as follows: (a) patient data from the Confusion Assessment Method for the ICU (CAM-ICU) or Richmond Agitation Sedation Scale (RASS) for delirium assessment were unavailable; (b) VA-ECMO was utilized; or (c) patients were of pediatric age (below 18 years of age) at the time of cannulation.

### Delirium screening

The CAM-ICU (19) has been widely used for assessing delirium (9) and is recommended for the screening of delirium in ICUs by the Society of Critical Care Medicine (20). As part of daily nursing management in the CVSICU, the CAM-ICU is completed every 4 h. Patients are concurrently assessed by the RASS (21) (a 10-point scale ranging from −5 [unarousable] to +4 [combative]). If a patient's RASS score is −5 (unarousable) or −4 (deep sedation), then that patient is deemed to be in a coma and unassessable for delirium using the CAM-ICU. If the patient is less sedated, with a RASS of −3 or greater, then the CAM-ICU assessment could be conducted for the presence of delirium. Three delirium subtypes were defined, using the patient's RASS score at the time of a positive CAM-ICU, as done in patients with COVID-19 in the ICU (15). These subtypes are (a) hyperactive, ranging from RASS of +1 (restless) to +4 (combative); (b) hypoactive, ranging from −3 (moderate sedation) to 0 (alert and calm); and (c) mixed, indicating periods of both hyper- and hypo-activity. Patients were also examined using the institutional Behavioral Pain Assessment Scale (BPAS), similar to the well-validated Critical-Care Pain Observation Tool (22) (CPOT) for non-verbal pain screening. The BPAS is a 10-point scale that evaluates five non-verbal signs of pain: facial expression, restlessness, muscle tone, vocalization, and consolability. Each sign is scored from 0 to 2, with a total score of 0, indicating no evidence of pain, 1–3 mild pain, 4–6 moderate pain, and 7–10 severe pain.

### Data collection and outcome measures

The Johns Hopkins University School of Medicine Institutional Review Board approved the current study (IRB00264320). Patient consent was waived due to the retrospective nature of the study. From the JHH-ECMO registry and the Epic Electronic Medical Record, we obtained information on each patient's demographics, pre/post-cannulation labs, relevant clinical information related to COVID-19 and ECMO management, delirium assessment data, and hospital course.

As part of routine practice at JHH, patients placed on VV-ECMO undergo non-invasive multimodal neuromonitoring, (23) consisting of an electroencephalogram, transcranial doppler, somatosensory evoked potentials, interval computed tomography (CT), and near-infrared spectroscopy. The neurocritical care team follows the patients to provide recommendations on these diagnostic modalities for each patient.

We electronically retrieved from the EMR CAM-ICU, RASS, and BPAS data on VV-ECMO days. We additionally obtained administration records of the following commonly used ICU medications for analgesia, sedation, agitation, and paralysis administered on VV-ECMO days—clonazepam, lorazepam, midazolam, propofol, oxycodone, fentanyl, hydromorphone, morphine, ketamine, dexmedetomidine, clonidine, quetiapine, risperidone, haloperidol, rocuronium, vecuronium, and cisatracurium. These records include medication name, concentration, mode of administration (intravenous [IV] injection, IV bolus from bag, continuous IV infusion, oral [PO] tablets, PO suspension, and patch), bolus or infusion doses, and the time interval of administration. This information was used to calculate a cumulative dose while on VV-ECMO, divided by the number of ECMO days to determine the average daily doses for each patient. The average dose per kilogram admission body weight was then calculated to adjust for patient weight. The weight- and non-weight-based averages do not include zero values for those who did not receive the medication. The IV midazolam-equivalent benzodiazepine dose was calculated using the conversion ratios of 1 mg IV midazolam to 0.25 mg PO clonazepam and 0.5 mg IV/PO lorazepam (24). The oral morphine-equivalent narcotic dose was calculated using conversion ratios of 30 mg PO morphine being equivalent to 100 μg IV fentanyl, 7.5 mg PO hydromorphone, 1.5 mg IV hydromorphone, and 20 mg PO oxycodone (24).

The primary outcomes were delirium prevalence and duration in the proportion of days on VV-ECMO. Total delirium days were counted by manual data review, where screening positive at least once a day would qualify as a delirium-positive day. The remaining days were either delirium-negative days or delirium-unassessable days. Additional days counted individually included (1) days on paralytics, (2) delirium-negative days off paralytics, and (3) days with unassessable CAM-ICU with a RASS of −4 or −5. Secondary outcomes included daily doses of analgosedatives and in-hospital outcomes. Right ventricular (RV) dysfunction was defined using an echocardiogram performed by clinicians at the bedside or by cardiologists for formal interpretations during the current hospitalization. Fatal intracerebral hemorrhage (ICH) was defined as one resulting in unsalvageable injury (i.e., brain herniation) and/or a clinical diagnosis of brain death.

### Statistical analysis

Statistical comparisons were performed between the survivors and non-survivors. Due to the relatively small subgroup size, median values with interquartile range (IQR) were reported for all continuous variables, with the non-parametric Wilcoxon Rank-sum test used to compare all continuous variables, regardless of data normality. Pearson chi-square analysis was used for categorical variables with all cell sizes of five or more patients. Fisher's exact test was used for variables with any cell having fewer than five patients. Spearman's rank correlation coefficients were calculated to assess for associations between either continuous or ordinal variables. An exploratory logistic regression analysis was conducted to examine the association between the proportion of delirium-positive days on VV-ECMO with mortality, adjusting for patient age, total days on ECMO, anti-interleukin-6 (IL-6) treatment, right ventricular dysfunction, secondary infection, and fatal ICH. All statistical analyses were conducted using STATA 17 (StataCorp. 2021. Stata Statistical Software: Release 17. College Station, TX: StataCorp LLC.) and GraphPad Prism (GraphPad Software, San Diego, CA USA, www.graphpad.com), with *p* < 0.05 set as the *a priori* criterion for statistical significance.

## Results

We identified a total of 47 patients on VV-ECMO for the treatment of COVID-19-related severe ARDS, of whom 35 (74%) were men and the median age was 51 (40, 55) years. Patient race/ethnicity consisted of White (*n* = 15), Hispanic/Latino (*n* = 16), African American (*n* = 14), and Asian (*n* = 2). Baseline demographics are summarized for the 21 (44.6%) survivors and 26 (55.3%) non-survivors in Table 1, with only a minority presenting with chronic cardiovascular comorbidities. Pre-cannulation complete blood count and arterial blood gas labs, APACHE II and SOFA scores, and other stratifying markers of COVID-19-related systemic inflammation (i.e., IL-6 and c-reactive protein) were comparable between the survivors and non-survivors. There was a higher proportion of non-survivors receiving anti-IL-6 treatment than the survivors (69.2 vs. 38.1%, *p* = 0.03). Similar proportions of survivors and non-survivors received the other treatments for COVID-19 and VV-ECMO, including prone positioning, airway pressure release ventilation (APRV), pressors, and anticoagulation.

**Table 1:** Baseline characteristics and clinical management of the severe COVID-19 ARDS patients on VV-ECMO between survivors vs. non-survivors.

**Characteristics**	**Survivors (*n =* 21)**	**Non-survivors (*n =* 26)**	***P*-value^a^**
Age^b^, yrs	47 (38, 53)	51.5 (45.8, 55)	0.13
Sex			
Female, *n* (%)	8 (38.1)	4 (15.4)	0.08
Male	13 (61.9)	22 (84.6)	
BMI^b^ (kg/m^2^)	32.5 (30.9, 39.4)	34.1 (29.1, 36.6)	0.60
Race and ethnicity			
African American, *n* (%)	8 (38.1)	6 (23.1)	0.15^d^
Hispanic/Latino	9 (42.9)	7 (26.9)	
White	4 (19.0)	11 (42.3)	
Asian	0 (0)	2 (7.7)	
Hypertension, *n* (%)	6 (28.6)	11 (42.3)	0.33
Hyperlipidemia, *n* (%)	2 (9.5)	8 (30.7)	0.15^d^
Diabetes, *n* (%)	7 (33.3)	7 (26.9)	0.63
Smoking history, *n* (%)	1 (4.8)	0 (0)	0.45^d^
Coronary artery disease, *n* (%)	0 (0)	3 (11.5)	0.24^d^
Pre-cannulation laboratory variables			
APACHE II Score^b, c^	17 (12, 22)	15 (11, 20)	0.46
SOFA Score^b, c^	8 (7, 10)	7 (8, 9)	0.78
Hemoglobin^b^ (g/dL)	11.4 (9.6, 12.3)	11.9 (10.9, 14.2)	0.59
WBC^b^ (10^3^ cells/mL)	15.3 (11.5, 18.9)	13.7 (12.2, 21.7)	0.96
Platelet^b^ (10^3^ cells/mL)	272 (176, 357)	223 (167, 292)	0.34
P/F ratio^b^ (mmHg)	63 (56, 67)	65 (55, 70.8)	0.65
PaO${}_{2}^{\text{b}}$ (mmHg)	63 (56, 67)	63 (54, 69.5)	0.99
PaCO${}_{2}^{\text{b}}$ (mmHg)	66 (61, 82)	64.5 (59, 75.3)	0.62
pH^b^	7.23 (7.15, 7.30)	7.27 (7.20, 7.34)	0.24
Lactate^b^ (mmol/L)	2.3 (1.5, 4)	2.3 (1.5, 2.8)	0.70
IL-6^b, e^ (pg/mL)	709.5 (172.2, 1,589)	267.1 (37.0, 1,082)	0.42
LDH^b^ (IU/L)	544 (406, 751)	564 (440, 637)	0.86
Ferritin^b, f^ (μ*g*/L)	1,071 (514, 1,859)	1,114 (755, 1,803)	0.30
CRP^b, f^ (mg/L)	11.9 (3.9, 31.9)	10.7 (4.7, 22.5)	0.62
COVID-19 VV-ECMO management			
ECMO day 1 PaO${}_{2}^{\text{b}}$ (mmHg)	77 (70, 92)	79.5 (66.5, 108.5)	0.97
ECMO day 1 PaCO${}_{2}^{\text{b}}$ (mmHg)	46 (40, 50)	47 (42.3, 53.5)	0.73
ECMO day 1 pH^b^	7.37 (7.35, 7.41)	7.39 (7.35, 7.42)	0.49
Proned, *n* (%)	20 (95.2)	26 (100)	0.45^d^
Inhaled nitric oxide, *n* (%)	20 (95.2)	22 (84.6)	0.36^d^
APRV ventilator mode, *n* (%)	12 (57.1)	12 (46.2)	0.45
Pressor requirement, *n* (%)	16 (76.2)	23 (88.5)	0.44^d^
Remdesivir, *n* (%)	12 (57.1)	21 (80.8)	0.08
Anti-IL-6 treatment, *n* (%)	8 (38.1)	18 (69.2)	0.03
Steroid treatment, *n* (%)	13 (61.9)	22 (84.6)	0.10^d^
Anticoagulation, *n* (%)	18 (85.7)	25 (96.2)	0.31^d^

APACHE II, the Acute Physiology and Chronic Health Evaluation II; APRV, airway pressure release ventilation; BMI, body mass index; CRP, C-reactive protein; ECMO, extracorporeal membrane oxygenation; IL-6, Interleukin-6; IQR, interquartile range; LDH, lactate dehydrogenase; P/F, P_a_O_2_/FiO_2_; SOFA, the Sequential Organ Failure Assessment; WBC, white blood cell. ^a^*p* < 0.05 was considered statistically significant. ^b^Values were presented as median (IQR). Wilcoxon rank-sum test was used. ^c^The SOFA and APACHE II scores were obtained 24 hohurs prior to cannulation. ^d^Fisher's exact test was used. ^e^IL-6 level was obtained in 31 patients. ^f^Ferritin and CRP levels were obtained in the same 41 patients.

Table 2 lists clinical characteristics of delirium while on VV-ECMO and additional information related to RASS scores and the use of analgosedatives and paralytics. Approximately all (97.5%) patients with assessable neurological exams in the survivor and non-survivor groups screened positive for delirium at some time point on VV-ECMO. The two patient groups initially screened positive for delirium after a similar duration of time on VV-ECMO [9.5 (5, 14) vs. 8.5 (5, 20) days after cannulation, *p* = 0.56]. Survivors and non-survivors also had similar total delirium-positive days, both measured as the number of days (*p* = 0.43) and as a proportion of days on VV-ECMO (*p* = 0.35). Every patient with delirium demonstrated the hypoactive subtype, with about half in each group also exhibiting a hyperactive component. In non-survivors, the median delirium day occurred significantly later since VV-ECMO cannulation compared to survivors (day 27.3 [17.4, 46.4] vs. 17 [9.9, 28], *p* = 0.04). The non-survivors also had significantly greater delirium-unassessable days on VV-ECMO with RASS of −4/−5 (23 [16.3, 38.3] vs. 17 (6, 22), *p* = 0.03) and total VV-ECMO days (44.5 [20.5, 74.3] vs. 27 [21, 38], *p* = 0.04). The delirium-unassessable days accounted for 53.1% (29.8–72.3) and 66.8% (44.5–90.0) of total VV-ECMO days for survivors and non-survivors, respectively. During their days on VV-ECMO, there were near-significant trends of lower BPAS and RASS results in the non-survivors, suggesting a deeper state of sedation (*p* = 0.054 and 0.06, respectively). All of the patients needed to be on at least one neuromuscular blocker, for a median of 10 (6–16) days, with survivors and non-survivors showing very few (1 [0, 3] vs. 0 [0, 3.8]) delirium-free days when off the neuromuscular blockade.

**Table 2:** Delirium characteristics while on VV-ECMO between survivors vs. non-survivors.

**Characteristics**	**Survivors (*n =* 21)**	**Non-survivors (*n =* 26)**	***P*-value^a^**
Delirium			
Present on CAM-ICU, *n* (%)	18 (85.7)	22 (84.6)	0.52^b^
Absent on CAM-ICU	1 (4.8)	0 (0)	
Unassessable with low RASS	2 (9.5)	4 (15.4)	
Delirium subtypes			
Hypoactive delirium, *n* (%)	10 (55.6)	10 (45.5)	0.55^b^
Hyperactive delirium	0	0	
Mixed delirium	8 (44.4)	12 (54.5)	
BPAS^c^	0.33 (0.16, 0.70)	0.15 (0.07, 0.42)	0.054
RASS^c^	−3.10 (−3.91, −2.21)	−3.72 (−4.42, −2.96)	0.06
Delirium onset since ECMO cannulation^c, d^ (days)	9.5 (5, 14)	8.5 (5, 21)	0.56
Median delirium days^c, d^ (days)	17 (9.9, 28)	27.3 (17.4, 46.4)	0.04
Total delirium days^c, d^ (days)	9.5 (3.3, 16.8)	9 (4.3, 28.3)	0.43
Proportion of total delirium days^c, d^ (%)	34.2 (18.3, 48.9)	24.8 (10.9, 43.8)	0.35
NMB days^c^ (days)	9 (4, 13)	12 (7, 20)	0.11
Proportion of NMB days^c, e^ (%)	27.7 (19.6, 50.0)	30.0 (18.6, 45.8)	0.91
Delirium-free days off NMB^c^ (days)	1 (0, 3)	0 (0, 3.8)	0.63
Proportion of delirium-free days off NMB^c, e^ (%)	2.9 (0, 18.4)	0 (0, 7.7)	0.29
Delirium-unassessable days with RASS of −4/−5^c^ (days)	17 (6, 23)	23 (16.3, 38.3)	0.03
Proportion of delirium-unassessable days^c, e^ (%)	53.1 (29.8, 72.3)	66.8 (44.5, 90.0)	0.18
ECMO duration^c^ (days)	27 (21, 38)	44.5 (20.5, 74.3)	0.04

BPAS, behavioral pain assessment scale; CAM-ICU, the Confusion Assessment Method for the Intensive Care Unit; ECMO, extracorporeal membrane oxygenation; IQR, interquartile range; NMB, neuromuscular blocker; RASS, Richmond Agitation Sedation Scale. ^a^*p* < 0.05 was considered statistically significant. ^b^Fisher's exact test was used. ^c^Values were presented as median (IQR). Wilcoxon rank-sum test was used. ^d^Delirium by CAM-ICU was detected only in 40 patients. ^e^The proportion is reported in percentage out of total ECMO cannulation days.

**Figure 1:**
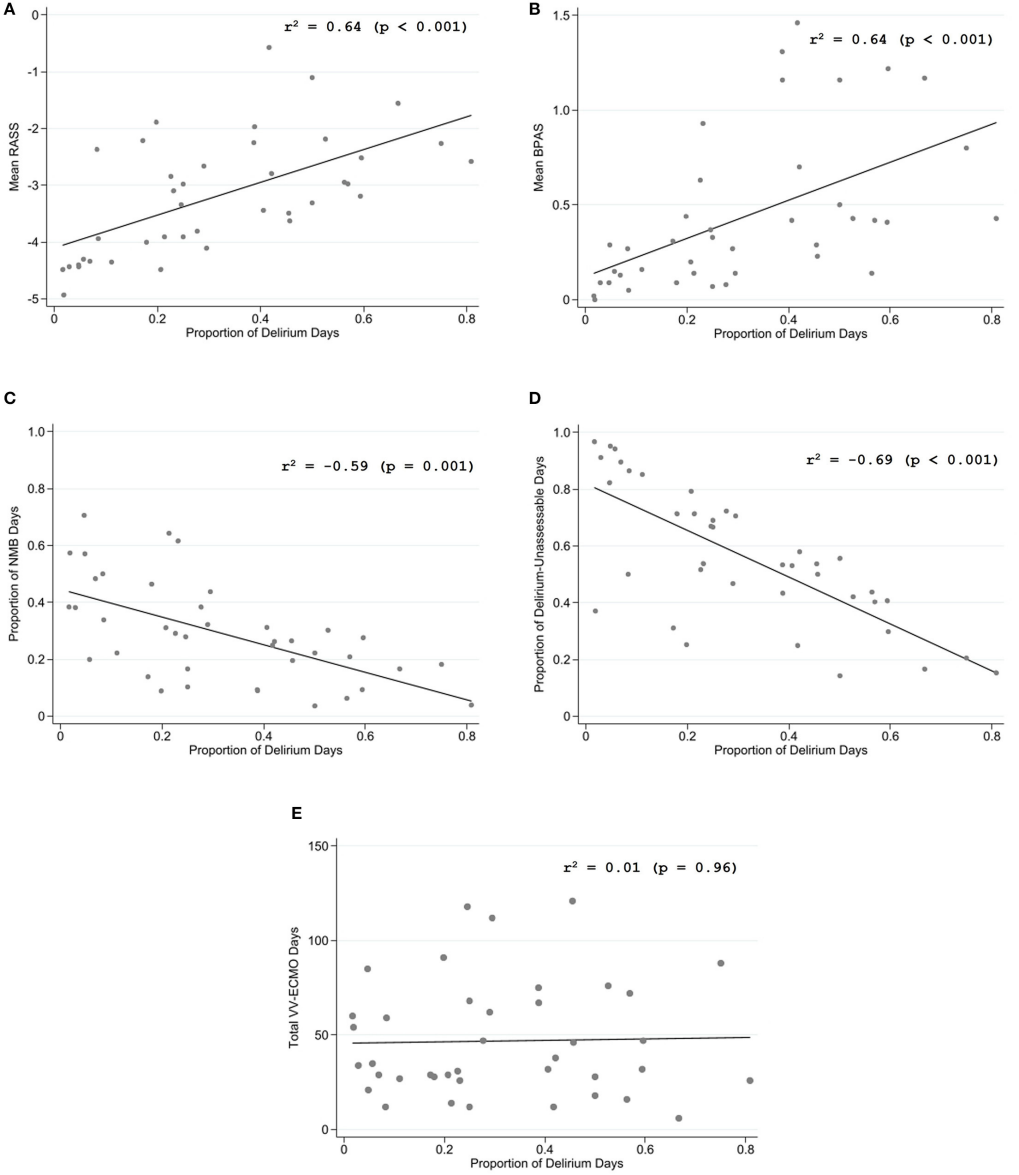
Scatterplots of Spearman's correlation between proportion of delirium days on VV-ECMO with **(A)** mean Richmond Agitation Sedation Scale (RASS), **(B)** mean Johns Hopkins behavioral pain assessment scale (BPAS), **(C)** proportion of days on neuromuscular blocker (NMB), **(D)** proportion of delirium-unassessable days, and **(E)** total days on VV-ECMO. BPAS, behavioral pain assessment scale; VV-ECMO, venovenous extracorporeal membrane oxygenation; NMB, neuromuscular blocker; RASS, Richmond Agitation Sedation Scale.

According to the Spearman correlation analysis (Table 3), the proportion of delirium-present days on VV-ECMO was moderately correlated with mean RASS (r = 0.64, *p* < 0.001) and mean BPAS (r = 0.64, *p* < 0.001), indicating more frequent detection of delirium on a lighter sedation level and a higher behavioral pain scale. The same time fraction entity of delirium was moderately inversely correlated with the proportions of days on a neuromuscular blocker (r = −0.59, *p* = 0.001) and the proportion of delirium-unassessable days (r = −0.69, *p* < 0.001). No correlations were found with age (r = −0.03, *p* = 0.88), total ventilator days (r = 0.04, *p* = 0.80), or overall ECMO duration (r = 0.01, *p* = 0.96). Scatterplots demonstrating the key correlations are depicted in Figure 1.

**Table 3:** Spearman correlation between proportion of delirium days and patient characteristics and in-hospital clinical outcomes in 40 patients with delirium.

**Variable**	**Spearman correlation coefficient (r)^a^**	***P*-value^b^**
Age (days)	−0.03	0.88
Admission APACHE II Score	−0.06	0.74
Admission SOFA Score	−0.12	0.45
Admission Lactate (mmol/L)	−0.11	0.50
Mean RASS	0.64	< 0.001
Mean BPAS	0.64	< 0.001
Proportion of delirium-unassessable days (%)	−0.69	< 0.001
Proportion of days on NMB days (%)	−0.59	0.001
ECMO duration (days)	0.01	0.96
Total ventilator days (days)	0.04	0.80
Length of hospitalization (days)	0.08	0.61

APACHE II, the Acute Physiology and Chronic Health Evaluation II; BPAS, behavioral pain assessment scale; ECMO, extracorporeal membrane oxygenation; IQR, interquartile range; NMB, neuromuscular blocker; RASS, Richmond Agitation Sedation Scale; SOFA, the Sequential Organ Failure Assessment. ^a^Coefficient (r) values >0 indicate a positive association; values < 0 indicate a negative association. ^b^*p* < 0.05 was considered statistically significant.

Table 4 lists the proportion of patients who received the reviewed analgosedatives, antipsychotics, and paralytic medications, as well as the body weight-adjusted and non-weight-adjusted mean daily dosages in each group. Of the searched medications, risperidone, haloperidol, rocuronium, and morphine were not used in any patients. Ketamine, hydromorphone, and vecuronium were used in every patient at some time point on VV-ECMO. Neither the frequency with which each medication was used nor the average daily dose, weight-adjusted or not, differed significantly between survivors and non-survivors. The median number of analgosedatives was seven in both groups, indicating a similar level of diversification. In addition to individual medication doses, the benzodiazepine and narcotic equivalents indicated comparable use of their cumulative doses and average dose per day.

**Table 4:** Weight-based and non-weight-based daily medication dosing average on VV-ECMO days between survivors vs. non-survivors.

**Medication^a^**	**Unit**	**Survivors (*n =* 21)**	**Non-survivors (*n =* 26)**	***P*-value^b^**
Clonazepam	*n* (%)	18 (85.7%)	26 (100%)	0.08^c^
	mg/day^d^	15.3 (9.9, 17.6)	14.0 (7.7, 17.7)	0.99
	mg/kg/day^d^	0.14 (0.09, 0.19)	0.13 (0.08, 0.18)	0.93
Lorazepam	*n* (%)	4 (19.0%)	6 (23.1%)	1^c^
	mg/day^d^	2.2 (0.2, 13.8)	1.72 (1.19, 3.17)	0.91
	μg/kg/day^d^	30.3 (2.1, 172)	20.7 (14.2, 29.2)	0.91
Midazolam	*n* (%)	17 (81.0%)	19 (73.1%)	0.73^c^
	mg/day^d^	6.2 (3.6, 14.0)	5.7 (2.1, 18.7)	0.83
	μg/kg/day^d^	59.1 (39.4, 130)	60.8 (23.1, 163)	0.93
Propofol	*n* (%)	18 (85.7%)	24 (92.3%)	0.64^c^
	mg/day^d^	165.7 (17.1, 440.6)	111.6 (25.0, 376.6)	0.99
	μg/kg/day^d^	1,647 (196, 4,921)	999.5 (257.2, 4,195)	0.99
Oxycodone	*n* (%)	17 (81.0%)	16 (61.5%)	0.20^c^
	mg/day^d^	111.6 (10.6, 181.7)	22.7 (0.9, 112.3)	0.19
	mg/kg/day^d^	1.09 (0.11, 2.17)	0.19 (0.01, 1.15)	0.15
Fentanyl	*n* (%)	16 (76.2%)	23 (88.5%)	0.44^c^
	μg/day^d^	242.2 (49.6, 659.6)	74.2 (39.7, 162.7)	0.13
	μg/kg/day^d^	2.79 (0.41, 7.24)	0.96 (0.43, 1.79)	0.15
Hydromorphone	*n* (%)	21 (100%)	26 (100%)	1^c^
	mg/day^d^	119.7 (76.3, 174.8)	154.6 (77.3, 208.5)	0.37
	mg/kg/day^d^	1.21 (0.83, 1.68)	1.34 (0.82, 2.16)	0.41
Ketamine	*n* (%)	21 (100%)	26 (100%)	1^c^
	mg/day^d^	1,332 (714, 1,539)	1,641 (827, 2,883)	0.27
	mg/kg/day^d^	13.0 (9.4, 16.9)	16.9 (8.6, 24.2)	0.28
Dexmedetomidine	*n* (%)	16 (76.2%)	18 (69.2%)	0.60
	μg/day^d^	268.8 (90.1, 705.1)	570.1 (201.1, 1,002.1)	0.24
	μg/kg/day^d^	2.61 (1.01, 6.50)	6.43 (1.91, 11.65)	0.20
Clonidine	*n* (%)	1 (4.8%)	2 (7.7%)	1^c^
	μg/day^d^	18.4	7.3 (6.5, 8.0)	0.67
	μg/kg/day^d^	0.16	0.073 (0.071, 0.076)	0.67
Quetiapine	*n* (%)	15 (71.4%)	18 (69.2%)	0.87
	mg/day^d^	34.2 (10.0, 88.0)	81.9 (31.7, 130.2)	0.24
	mg/kg/day^d^	0.30 (0.10, 0.89)	0.80 (0.40, 1.35)	0.20
Vecuronium	*n* (%)	21 (100%)	26 (100%)	1^c^
	mg/day^d^	30.7 (13.0, 52.1)	37.2 (21.2, 78.1)	0.29
	μg/kg/day^d^	272 (150, 474)	416 (206, 639)	0.23
Cisatracurium	*n* (%)	4 (19.0%)	4 (15.4%)	1^c^
	μg/day^d^	7,635 (4,534, 41,126)	335 (282, 13,263)	0.40
	μg/kg/day^d^	86 (51, 380)	4.6 (3.7, 166.8)	0.40
Total number of analgo-sedatives^e^	*n*	7 (6, 8)	7 (7, 8)	0.80
Benzodiazepine equivalent^f^	total^d^ (mg)	934 (484, 1,533)	1,395 (904, 2,775)	0.10
	mg/day^d^	34.6 (28.2, 42.2)	33.6 (21.4, 56.1)	0.77
	mg/kg/day^d^	0.32 (0.26, 0.45)	0.42 (0.18, 0.48)	0.86
Narcotic equivalent^g^	total^d^ (kg)	1.18 (0.40, 2.59)	1.36 (0.36, 2.32)	0.92
	g/day^d^	19.6 (10.6, 140)	24.1 (10.7, 49.4)	0.72
	mg/kg/day^d^	228 (94, 1,528)	290 (129, 464)	0.74

IQR, interquartile range; IV, intravenous; PO, per os (by mouth). ^a^Only intravenous, patch, or oral formulations were included. Epidural administrations were excluded as their primary effect is local anesthesia. ^b^*p* < 0.05 was considered statistically significant. ^c^Fisher's exact test was used. ^d^Values were presented as median (IQR). Wilcoxon rank-sum test was used. The weight-based and non-weight-based averages do not include zero values for those who did not receive the medication. ^e^Includes clonazepam, lorazepam, midazolam, propofol, oxycodone, fentanyl, hydromorphone, ketamine, dexmedetomidine, and clonidine. ^f^Intravenous midazolam-equivalent benzodiazepine dosing was calculated by the following conversion ratios: 1 mg IV midazolam is considered equivalent to 0.25 mg PO clonazepam and 0.5 mg IV/PO lorazepam. ^g^Oral morphine-equivalent narcotic dosing was calculated by the following conversion ratios: 30 mg PO morphine is considered equivalent to 100 μg IV fentanyl, 7.5 mg PO hydromorphone, 1.5 mg IV hydromorphone, and 20 mg PO oxycodone.

Medical complications relating to the management of severe COVID-19 ARDS are reviewed in Table 5. The timing of ECMO cannulation since intubation, use of tracheostomy, the timing of tracheostomy placement since ECMO cannulation, total ventilator days, and hospital days were similar in survivors and non-survivors. The non-survivors had a higher rate of right ventricular dysfunction (50.5 vs. 14.3%, *p* = 0.01). While the overall rate of ICH was similar, found in four of 21 (19.0%) survivors vs. in seven of 26 (26.9%) non-survivors, five of the seven ICH were fatal and caused demise in the non-survivor group. Two of 26 (7.7%) who did not have fatal ICH were withdrawn from their life-sustaining support by family. Three (11.5%) other patients died after decannulation: two from post-decannulation sepsis and one from a bronchopleural fistula. The other complications (i.e., systemic hemorrhage and secondary infection) occurred similarly in survivors and non-survivors. An exploratory multivariate logistic regression analysis, adjusting for patient age, secondary infection, and all discrepant findings between non-survivors and survivors—days on ECMO, fatal ICH, anti-IL-6 treatment, and RV dysfunction—indicated no association between the proportion of delirium-positive days and mortality (*p* = 0.13).

**Table 5:** In-hospital ventilator management and complications of the severe COVID-19 ARDS patients on VV-ECMO between survivors vs. non-survivors.

**Characteristics**	**Survivors (*n =* 21)**	**Non-survivors (*n =* 26)**	***P*-value^a^**
ECMO cannulation since intubation^b^ (days)	4 (2, 5)	4.5 (2, 6)	0.57
Tracheostomy, *n* (%)	18 (85.7)	25 (96.2)	0.31^c^
Tracheostomy placement since ECMO cannulation^b^ (days)	14 (10.5, 16)	11 (8, 14)	0.17
Total ventilator days^b^ (days)	49 (30, 59)	48.5 (23.3, 83.3)	0.55
Length of hospitalization^b^ (days)	60 (43, 80)	45 (21.5, 86.3)	0.35
Complications			
RV dysfunction, *n* (%)	3 (14.3)	13 (50.0)	0.01^c^
AKI, *n* (%)	7 (33.3)	10 (38.5)	0.72
Secondary infection, *n* (%)	18 (85.7)	20 (76.9)	0.71^c^
Bleeding, *n* (%)	15 (71.4)	23 (88.5)	0.26^c^
ICH, *n* (%)	4 (19.0)	7 (26.9)	0.73^c^
Fatal ICH^d^, *n* (%)	0 (0)	5 (19.2)	0.056^c^
Thrombosis, *n* (%)	9 (42.9)	5 (19.2)	0.11^c^

AKI, acute kidney injury; ECMO, extracorporeal membrane oxygenation; ICH, intracerebral hemorrhage; IQR, interquartile range; RV, right ventricle. ^a^*p* < 0.05 was considered statistically significant. ^b^Values were presented as median (IQR). Wilcoxon rank-sum test was used. ^c^Fisher's exact test was used. ^d^Fatal intracerebral hemorrhage was defined as those resulting in unsalvageable injury (i.e., brain herniation) and/or clinical diagnosis of brain death.

## Discussion

Delirium, despite its common occurrence in the ICU, remains ineffectively prevented and treated due to its multifactorial etiology and poorly understood pathophysiology. Our study confirms that the condition is nearly ubiquitous in severe COVID-19 ARDS patients requiring VV-ECMO who have assessable neurological examinations. Furthermore, those who ultimately survived and those who did not, similarly, spent the majority of their VV-ECMO days heavily sedated or comatose, thereby limiting the role of delirium to represent the overall neurological status in our patients. Paralytics were also required in every patient in our cohort, as was proning for nearly all patients prior to cannulation to treat COVID-19 severe ARDS. With these provided restrictions, we utilized the proportion of days on VV-ECMO with delirium as a normalized measure to demonstrate that delirium was found more often when the patients were more lightly sedated, had more pain, and had fewer days in a coma or on the neuromuscular blockade. In this very sick population with both survivors' and non-survivors' mean RASS scores of below −3 (moderate sedation), delirium characteristics were mostly similar in proportion to ECMO duration, and the detected duration of delirium did not predict mortality in our multivariable logistic regression analysis.

Our critically ill patients expectedly had a common occurrence of delirium, a well-known predictor of poor outcomes in the ICU (2, 4, 8, 10). Our finding in a relatively young cohort is consistent with a recent large-scale study on delirium in acute heart failure patients, in which delirium was similarly common in patients younger than 65 years old (10). This highlights the importance of managing the underlying acute medical illness even in younger and healthier patients with a good cognitive reserve. Non-survivors, despite having comparable delirium characteristics and dosing of analgosedatives, antipsychotics, and paralytics, ended up being on VV-ECMO longer with more delirium-unassessable days. We found that non-survivors had trended toward lower RASS scores, closer to −4 (deep sedation), in the setting of the requirement of prolonged ECMO therapy. Their poorer neurological status can be related to the complicated medical course from severe COVID-19 (i.e., ARDS requiring anti-IL-6 therapy), acute cardiac dysfunction (10), fatal ICH, and iatrogenic from heavy sedation, ventilator, and ECMO therapies. The authors believe that the secondary infection and ICH variably contributed to delirium and mortality in individual patients despite their comparable occurrences in survivors and non-survivors. Incorporation of standardized neuromonitoring protocols [i.e., non-pharmacological interventions for delirium prevention (25) and multimodal neuromonitoring protocols (23)] into the ECMO therapy starting early in their hospital course, when feasible, should facilitate diagnosis and treatment in these critically ill patients.

Patients with COVID-19 in the ICU are known to have higher sedation requirements, likely related to younger age with fewer chronic comorbidities, high respiratory drive, and intense inflammatory responses linked to tolerance (26). The authors believe that our analgosedation strategy was an effective way to manage severe COVID-19 ARDS, especially early in the hospital course. However, it inevitably clouded the patient's neurological status. A reasonable interpretation of our findings is that less level of sedation (higher RASS) exacerbates pain and discomfort (i.e., patient-ventilator dyssynchrony), which can worsen delirium severity. Another explanation is that heavier levels of sedation mask clinical manifestations of delirium, as suggested by the similar total delirium days but the delayed median delirium days in the non-survivors over their more protracted ECMO therapy. While the mechanistic role of delirium here is difficult to elucidate, promoting lighter sedation may lead to improved neurological outcomes and reduced mortality. In two previously reported multicenter, observational studies, heavy sedation was found to predict mortality but was not associated with delirium, which occurred in 51 and 44% of patients (11, 13). Another study, for which the incidence of delirium was not reported, also identified a significant association between heavy sedation and mortality (12). According to the Extracorporeal Life Support Organization, sedation to the point of light anesthesia should be the goal during cannulation and all sedation and opioids should be stopped for interval neurological exams (27). The elevated medication clearance rate while on ECMO and the need to achieve ventilator synchrony, optimize ECMO flows, lower metabolic demand, and early liberation (28) make it difficult to lighten sedation.

There is no standard analgosedation practice for patients with COVID-19 on VV-ECMO. In a recent publication on patients with COVID-19 receiving VV-ECMO for predominantly moderate to severe ARDS, higher doses of ketamine, benzodiazepine, propofol, and dexmedetomidine, and less use of opioids were noted over the first 7 days compared to ours over the full course of VV-ECMO therapy (29). There is emerging evidence related to ECMO, such as the reduced dose of hydromorphone use due to its lower affinity to the ECMO circuit compared to fentanyl (27, 30). More studies are needed to better understand the pharmacokinetic properties of commonly used agents over the course of ECMO therapy. We performed exploratory, linear regression analysis between RASS scores and the daily median doses of commonly used medications while on VV-ECMO, and medications appear to have variable effects on sedation (Supplementary Table S1). Further studies are needed to validate analgosedation strategies [i.e., awake VV-ECMO strategy (31), use of adjunct agents (29), and patient-specific factors to consider in ECMO (27, 32)], as well as the implementation of novel, practical management approaches for the unstable patients [i.e., multimodal neuromonitoring (23) and bedside brain MRI (33)].

Our study results should be interpreted with caution. It is limited by its retrospective, observational design, which is subject to unmeasured confounding effects. We only included a specific population of patients with COVID-19 requiring VV-ECMO support, thus, our findings are not generalizable to other ECMO populations (i.e., venoarterial ECMO). On the other hand, there was a consistency in the profile of analgosedatives and multimodality analgesia per the institution's protocol. For instance, ketamine has not been shown to effectively prevent delirium (3, 5), but it was used in all our patients, given its safety profile and known benefits for hemodynamic support. The BPAS and RASS scores differed by < 1 point between survivors and non-survivors, and the differences were likely of unclear clinical significance in daily clinical decision-making. Due to the small sample size, our statistical analyses between survivors and non-survivors mainly remain descriptive without variable adjustments. Correlation does not imply causation, and the authors acknowledge that our interpretations based on clinical reasoning may not hold true. There were certain factors (i.e., mean RASS and fatal ICH) that showed near-significant trends between survivors and non-survivors, and although the study is not primarily designed to compare them, having a larger sample size could have resulted in statistical significance. Details on ventilator and ECMO management, withdrawal of care, and post-discharge outcomes were not captured in this study. Utilization of alternative outcome scales, such as the CAM-ICU-7 (15) and the Intensive Care Delirium Screening Checklist (34), could provide additional details on delirium that may guide clinical management.

## Conclusion

In our patients with severe COVID-19 ARDS on VV-ECMO, delirium was common. Its clinical manifestations appear to be influenced by the patient's level of sedation and time on paralytics and in a comatose state, and delirium duration did not independently predict in-hospital mortality. Despite being on comparable doses of analgesia and paralytics, non-survivors had poorer levels of wakefulness, attributed to a more complicated hospital course. Fatal ICH was a notable complication; as such, scheduled neurological examinations of sedation and multimodal bedside neuromonitoring might allow for earlier detection and treatment. Finally, further studies are needed to optimize analgosedation and paralytic strategies to improve delirium prevention and patient outcomes.

## Data availability statement

The raw data supporting the conclusions of this article will be made available by the authors, without undue reservation.

## Ethics statement

The studies involving human participants were reviewed and approved by Johns Hopkins University School of Medicine Institutional Review Board. Written informed consent for participation was not required for this study in accordance with the national legislation and the institutional requirements.

## Author contributions

PS: acquisition of data, analysis and interpretation of data, and drafting and revising the manuscript. JF: acquisition of data and drafting and revising the manuscript. AP, BS, JL, and GC: acquisition of data and revising the manuscript. GW, SC, BK, and S-MC: conception and design, analysis and interpretation of data, and drafting and revising the manuscript. All authors contributed to the manuscript and approved the submitted version.
