# Supplementary material for: Characteristics of delirium and its association with sedation and in-hospital mortality in patients with COVID-19 on veno-venous extracorporeal membrane oxygenation
Source: Front Med (Lausanne). 2023 May 25;10:1172063. doi: 10.3389/fmed.2023.1172063 (PMC10248255; doi:10.3389/fmed.2023.1172063)
Supplement: Supplementary file 1 [file Table_1.docx]

Table S1: Correlation between RASS and median daily dosage of ICU medications

| Medication^a^ | Number of patients (n) | Correlation coefficient (r)^b^ | p-value^b^ |
| --- | --- | --- | --- |
| Clonazepam (mg/d) | 44 | -0.07 | 0.001 |
| Lorazepam (mg/d) | 10 | -0.029 | 0.28 |
| Midazolam (mg/d) | 36 | 0.0045 | 0.66 |
| Propofol (μg/d) | 42 | -0.0055 | 0.18 |
| Oxycodone (mg/d) | 33 | -0.0021 | 0.36 |
| Fentanyl (μg/d) | 39 | 0.00045 | 0.050 |
| Hydromorphone (mg/d) | 47 | -0.0046 | 0.004 |
| Ketamine (mg/d) | 47 | -0.00049 | 0.000 |
| Dexmedetomidine (μg/d) | 34 | -0.000020 | 0.93 |
| Clonidine (μg/d) | 3 | 10.5 | 0.94 |
| Quetiapine (mg/d) | 33 | -0.0032 | 0.06 |
| Vecuronium (mg/d) | 47 | -6.6 * 10^-6^ | 0.002 |
| Cisatracurium (mg/d) | 8 | -8.6 * 10^-6^ | 0.52 |
| Benzodiazepine equivalent^c^ (mg/d) | 47 | -0.0086 | 0.06 |
| Narcotic equivalent^d^ (mg/d) | 47 | 1.7 * 10^-6^ | 0.023 |

Abbreviations: ICU, intensive care unit; IQR, interquartile range; IV, intravenous; PO, per os (by mouth); RASS, Richmond Agitation Sedation Scale

^a^ Only IV, patch, or oral formulations were included. Epidural administrations were excluded as their primary effect is local anesthesia. The daily dosing does not include zero values for those who did not receive the medication.

^b^ Coefficient (r) values > 0 indicate a positive association; values < 0 indicate a negative association. p < 0.05 was considered statistically significant.

^c^ Intravenous midazolam-equivalent benzodiazepine dosing was calculated by the following conversion ratios: 1mg IV midazolam is considered equivalent to 0.25mg PO clonazepam and 0.5mg IV/PO lorazepam.

^d^ Oral morphine-equivalent narcotic dosing was calculated by the following conversion ratios: 30mg PO morphine is considered equivalent to 100$\mu$g IV fentanyl, 7.5mg PO hydromorphone, 1.5mg IV hydromorphone, and 20mg PO oxycodone.
